# Acute physiological response to different recreational team handball game formats in over 60-year-old inactive men

**DOI:** 10.1371/journal.pone.0275483

**Published:** 2022-10-13

**Authors:** Ivone Carneiro, Peter Krustrup, Carlo Castagna, Rita Pereira, Eduardo Coelho, Susana Póvoas

**Affiliations:** 1 Research Center in Sports Sciences, Health Sciences and Human Development, CIDESD, University of Maia, ISMAI, Maia, Portugal; 2 Department of Sports Science and Clinical Biomechanics, SDU Sport and Health Sciences Cluster (SHSC), University of Southern Denmark, Odense, Denmark; 3 Danish Institute for Advanced Study (DIAS), University of Southern Denmark, Odense, Denmark; 4 Sport and Health Sciences, University of Exeter, Exeter, United Kingdom; 5 Shanghai University of Sport (SUS), Shanghai, China; 6 University of Roma Tor Vergata, Rome, Italy; 7 Laboratory of Metabolism and Exercise (LaMetEx), Research Centre in Physical Activity, Health and Leisure (CIAFEL), Faculty of Sport, University of Porto, Porto, Portugal; 8 University of Maia, ISMAI, Maia, Portugal; 9 Porto Sports Medicine Center (IPDJ, IP), Porto, Portugal; Sport Sciences School of Rio Maior - Politechnic Institute of Santarem, PORTUGAL

## Abstract

This study described the physical and physiological demands, activity profile and fun levels of recreational team handball (TH) game formats in over 60-year-old men with no previous experience with this sport (n = 17, 67.4±3.3 years). The participants performed 5v5, 6v6 and 7v7 matches (3x15-min periods) with fixed pitch size (40x20 m). In all testing sessions, heart rate (HR), differential ratings of perceived exertion and blood lactate were evaluated to measure internal load. Locomotor profile, game actions and accelerometer data were used to access external load. Also, fun levels were registered at the end of all testing sessions. Mean (76–77%HR_max_) and peak HR (84–86%HR_max_) decreased from the first to the third match period, in 6v6 and 7v7 (*p≤*0.034, *d =* 0.730). Blood lactate increased from baseline to the first period and decreased from the first to the third period in all game formats (*p<*0.001, *d =* 1.646). The participants covered longer total distances in 6v6 vs 5v5 (*p≤*0.005, *d =* 0.927) and spent more time in fast running in 6v6 vs 5v5 and 7v7 (*p<*0.001, *d =* 1.725) and in 5v5 vs 7v7 (*p =* 0.007, *d =* 0.912). A higher number of throws was performed in 5v5 vs 6v6 and 7v7 (*p<*0.001, *d =* 1.547), and in 6v6 vs 7v7 (*p =* 0.031, *d =* 0.779). The number of stops and total actions in 7v7 was significantly lower vs 5v5 and 6v6 (*p≤*0.003, *d =* 1.025). Recreational TH is a high-intensity and motivating exercise mode for middle-aged and older men, regardless the game format. However, higher high-intensity demands were observed during 5v5 and 6v6 game formats. Therefore, it is suggested a multiple game format (5v5, 6v6 and 7v7) training plan, with more use of 5v5 and 6v6 game formats, with training sessions lasting up to 15-min of warm-up and 3x15-min periods of match-play, when prescribing recreational TH to improve cardiovascular and musculoskeletal health in this population.

## Introduction

As world population’ life expectancy is increasing, promoting healthy behaviors and high health-related quality of life at old age, has become a major concern. Physical inactivity is a risk factor for several noncommunicable diseases and a leading cause of death for global mortality [1]. Men have higher incidence and death rates of ischemic heart disease, diabetes mellitus during midlife, and of most cancers (not related to reproduction) than women [2, 3]. On the other hand, the health benefits of physical activity (PA) and exercise for older adults are well established [4, 5]. Those include decreasing the prevalence of common chronic diseases, namely, those previously mentioned [6], and cognitive decline [7], increasing physical function, mental health [8] and, consequently, improving quality of life [9]. Nonetheless, 58% of the European middle-aged and older men (over 55 years old) do not exercise or play any sports, mainly due to lack of time or motivation [10]. Although the benefits of exercising outweigh the risks associated with being physically inactive [11], there is still a concern with the risk of injury [12]. This especially in older, inactive and exercise/sport inexperienced populations. Consequently, finding alternative exercise programs that are effective, safe, and motivating enough to ensure long-term adherence to exercise for this population is essential.

Exercise programs using recreational team sports, an adaptation of the official versions played as different small-sided games (SSGs), have been adopted as a motivating strategy to decrease physical inactivity and promote broad-spectrum health, physical fitness and well-being improvements in different populations [13–17]. Recreational team sports have shown to have a major beneficial impact on cardiorespiratory fitness [13, 15], which has been associated with the time spent with high heart rates (HR) during recreational team handball (TH) matches [18]. Despite the high physical and physiological demands imposed by SSGs, the participants have reported moderate ratings of perceived exertion (RPE) [19].

TH is played by around 30 million of players worldwide [20] being a particularly popular sport in Europe, namely in Portugal. If we add to this, the number of fans and supporters, the social capital emerging from this sport practice can be considered of great interest. Recreational TH has shown to be a high-intensity intermittent exercise mode [15, 21], effective in improving physical fitness and cardiometabolic health (e.g. maximal oxygen uptake (VO_2max_), blood pressure, aerobic performance, and blood lipid profile) in adult/middle-aged male former TH players [18], premenopausal overweight women [22], postmenopausal women [23] and young adult men [24] with no experience with the sport. In addition, it has proved to induce positive musculoskeletal adaptations (e.g. muscle mass, bone mineral content and density, and on bone metabolism) in young adult men [24] and women [25], and also in postmenopausal women [26].

SSGs are often used in recreational team sports exercise interventions as training tools [14–17, 19]. They are characterized by adapted rules compared to the official ones, such as number of players, size and shape of the court, allowed body contact, coach encouragement, among others, that influence internal and external load markers [27–29]. Recreational TH as exercise mode has been implemented using different game formats, ranging from formal (7v7) to 3v3 formats [15]. Notwithstanding the reported health benefits, no conclusive information exists on what is the most effective recreational TH game format to induce the reported adaptations. This issue is of great practical interest in the daily practice, as different number of participants may attend the training sessions, and also to guide future exercise interventions.

In competitive soccer, the number of players per playing surface has been reported to impact the game demands [30]. On other hand, in recreational soccer, high HRs were observed for different age, sex and social background groups, by playing SSGs, independently of number of players [31]. Additionally, similar physical and physiological demands were reported for 21-year-old college students during recreational TH SSGs (4v4, 5v5 and 6v6) [32]. Nevertheless, the demands of this exercise mode have not yet been described for older populations, and the specific demands of other game formats frequently used in recreational TH-based exercise interventions (i.e., 5v5, 6v6 and 7v7) are still to be ascertained.

In order to induce cardiovascular health improvements, average exercise intensity should range between 60–85%HR_max_ [33], and for optimal improvements it appears to be important to spend a significant amount of time of the training session with HRs above 85%HR_max_ [34]. In fact, in a 12-week recreational TH intervention, post-intervention changes in VO_2max_ were largely correlated with the time spent with HRs >90%HR_max_ [18]. Recreational team sports intensities have shown to be in the range of these intensities in different populations [15]. Nonetheless, this has not yet been described for middle-aged and older men. To optimize training load during recreational TH interventions, it is also important to describe the intensity of the different time periods within a proposed SSG. This to ascertain if a high intensity is maintained throughout the entire training session. Despite the interest of the maintenance of an effective exercise intensity during the matches, recreational TH internal and external load differences between match periods have only been addressed in one study with adult/middle-aged men with previous experience in TH [21]. In that study similar cardiovascular load during the entire match duration (60 min) was reported. However, a decrease was observed in the second half in the frequency and distance covered in some of the locomotor categories, specific game actions and blood lactate (BL) values [21]. Unfortunately, no study is currently available on the effects of game format duration on exercise intensity in over 60-year-old inactive men with no previous experience with recreational TH.

Long-term adherence to the exercise programs is a major concern when planning exercise interventions [35]. Recreational team sports have been considered as a social, fun and intrinsically motivational exercise mode [36], which are important characteristics for long-term adherence to exercise, namely, in the elderly male population [36]. Therefore, it is of relevance to evaluate the self-reported fun levels (which reflect enjoyment) during recreational TH played as different game formats, as it may well be a positive affective response and an intrinsically motivation factor for the participation and adherence of an individual to an exercise program [36]).

Given the above reported premises, the aim of this study was to describe the acute physiological response, activity profile and fun levels of 5v5, 6v6 and 7v7 recreational TH game formats in over 60-year-old men when played over the official court (40x20 m). We hypothesized that 5v5 elicits higher cardiovascular (internal load) and activity profile (external load) demands due to the larger playing area, and, consequently, lower player density, and higher fun levels, as a result of higher involvement of the participants in the match.

## Materials and methods

### Participants

The recruitment process was done through advertisement in social media (Facebook), flyers/posters, and face-to-face meetings in local senior institutions. Seventeen male participants (67.4±3.3 (±SD) years; stature 168.2±5.5 cm; body mass 79.0±11.8 kg; fat mass 29.0±6.2%; body mass index 27.8±3.2 kg⋅m^−2^; peak oxygen uptake (VO_2peak_) 27.9±4.1 mL⋅min^-1^⋅kg^−1^; systolic blood pressure 132±20 mmHg; diastolic blood pressure 78±8 mmHg; resting HR 69±12 b⋅min^−1^; Yo-Yo intermittent endurance level 1 test (YYIE1) 480±256 m) with no previous experience with this sport, agreed to participate in this study. Inclusion criteria were: male participants, aged over 60 years, inactive (i.e., not complying with the PA guidelines for the last 6 months). Exclusion criteria were: participants with medical contraindications to perform moderate-to-vigorous PA or incapacity to run or grip a ball. All the participants were informed about the study purposes, risks and benefits and signed a written informed consent according to the Declaration of Helsinki. Ethical approval was provided by the local Institutional Review Board (CEFADE 19 2019).

### Experimental design

**Fig 1:**
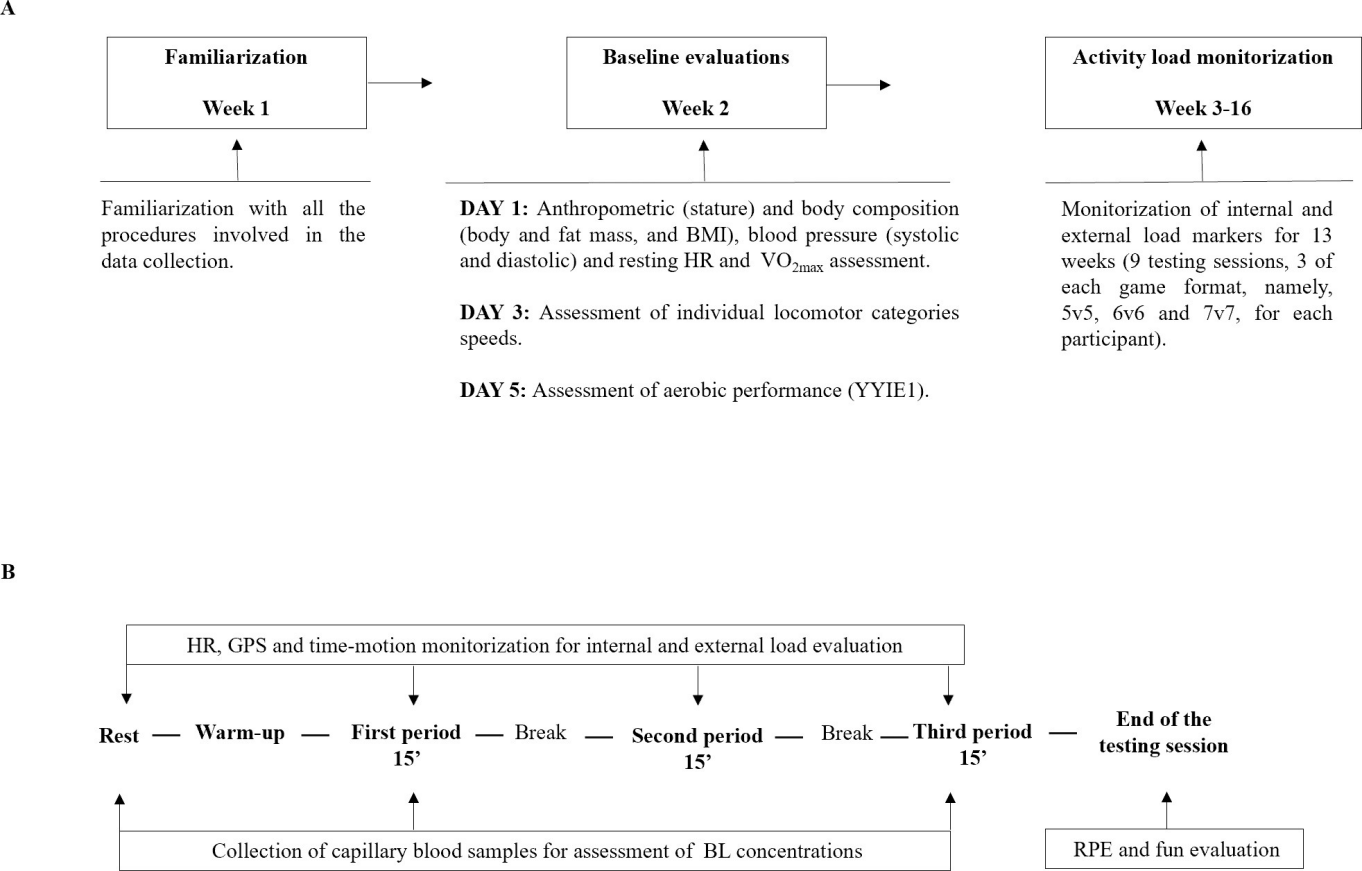
Schematic representation of the study protocol. BMI–Body mass index; HR–Heart rate; VO_2max_−Maximal oxygen uptake; YYIE1—Yo-Yo intermittent endurance level 1 test; GPS—Global positioning system; BL–Blood lactate; RPE—Rating of perceived exertion.

All the participants were familiarized with the procedures involved in this study in the week preceding the data collection. Evaluations started with assessment of anthropometric variables, body composition, blood pressure, resting HR and VO_2max_, in this order. Afterwards, on 2 separate days, the participants were tested for individual locomotor categories speed thresholds by performing each locomotor category at their individual speed, twice, over a 20 m distance, with 90-s recovery in-between, and for aerobic performance (YYIE1). Finally, internal and external load markers were monitored for each participant during 9 testing sessions. These sessions consisted of a standardized warm-up followed by recreational TH matches, 3 of each game format (5v5, 6v6 and 7v7), performed in a random order (Fig 1). These game formats were selected as they are typically used in recreational TH interventions that have shown to result in health improvements [15]. There were 48 hours between each testing session and the participants were asked to refrain from intense PA in the 48h before the testing sessions. The court size was 40x20 m, resulting in ~80, 67 and 57 m^2^ per player for 5v5, 6v6 and 7v7 game formats, respectively, to test the effect of player density.

Players’ internal load was evaluated as exercise HR, BL concentration and differential RPE. Fun levels were also recorded at the end of all testing sessions. TH high-demanding game actions (i.e., jumps, throws, changes of direction, one-on-one situations and stops) and distances covered in selected locomotion categories were considered to profile participants’ external load. With the aim to account for inter-individual variability in external load, time-motion analysis was performed according to participants’ individual speed categories.

All matches were performed during morning sessions and the participants wore t-shirts and shorts. The participants were hydrated at the beginning of the testing session and were allowed to drink water *ad libitum* to ensure the maintenance of proper hydration throughout the testing sessions. Each testing session comprised a 15-min standardized warm-up, consisting of running, coordination, flexibility, balance, and strength exercises, and three 15-min periods of recreational TH matches played either as 5v5, 6v6 and 7v7, interspersed by 2-min breaks.

The warm-up started with back-and-forth progressive intensity runs in the TH court combined with articular movements for the upper and lower body during approximately 5 min. Then, the other 10 min aimed at flexibility and balance exercises for the upper and lower body and at strength exercises for the main muscles, namely, squats, frontal and side lunges, push-ups, and frontal and side planks. The mean HR during the warm-up of the testing sessions was 69%HR_max_.

After the warm-up, the second part of the testing sessions consisted of three 15-min periods of recreational TH matches. At every 3-min during the matches, the participants were instructed to change their positions assuring even rotation between the participants in the outfield and goalkeeper positions. There were no players’ substitutions during the matches and the participants were instructed to follow the basic TH rules. However, the balls used were smaller (47 cm circumference, GOALCHA, Fredericia, Denmark) and made of softer material than the official TH balls, and no body contact was allowed. This, to avoid injuries, since the participants had no experience with this sport. Only data from participants that performed all the three 15-min periods were analyzed.

All testing sessions were instructed by a professional TH coach and physical education teacher and monitored by the research team. All the data collection and analysis were performed by the research team that comprised an experienced group of Sport Science, Physical Exercise and Health and Physical Education Teaching Master and PhD graduates, that had at least 5 years of experience with the testing procedures and analysis.

### Experimental procedures

#### Anthropometric and health outcomes procedures

Body mass (0.01 kg) and fat mass (%) were measured in a bioimpedance digital scale (Tanita Inner Scan BC 532, Tokyo, Japan) and stature (0.1 cm) was determined using a portable stadiometer (Seca 213, Hamburg, Germany), according to standardized protocols [37]. Body mass index was calculated (kg·m^−2^). Blood pressure and resting HR measurements were assessed with an automatic upper arm blood pressure monitor (multiparameter patient monitor, Omron Z207, Kyoto, Japan). The participants were required to sit and rest for at least 5 min prior to the first blood pressure measurement. Two measurements were taken after 5 and 10 min of rest from the right arm, with the participants seated, in a relaxed position with their feet resting flat on the ground. The mean of the two measurements was considered for blood pressure analysis. If the two measurements differed by 2 mmHg or more, a third measure was taken. The lowest resting HR value was considered for analysis [38].

To access VO_2max_, the participants performed an incremental treadmill test until voluntary exhaustion (H/P/Cosmos, Quasar, Germany) [39]. For this purpose, the participants walked on the treadmill for at least 3 min for each stage. The participant’s HR was taken every min, and if the participant’s HR was not at steady state by the 3^rd^ min, the test continued at that same stage for another min. The first stage was considered the warm-up stage and was performed at 2.7 km⋅h^-1^ and 10% inclination, the second stage at a 4 km⋅h^-1^ and 12% inclination, the third stage at a 5.4 km⋅h^-1^ and 14% inclination, the fourth stage at 6.7 km⋅h^-1^ and 16% inclination and the fifth stage at 8 km⋅h^-1^ and 18% inclination [39]. The test was performed until voluntary exhaustion. The participants completed at least all the three first stages and the fifth stage was the highest reached. VO_2max_ and respiratory exchange ratio (RER) were determined by pulmonary gas exchange measurements (Oxycon Pro Metabolic Cart, Jaeger, careFusion, Germany) with the participants wearing a HR monitor (Polar Wearlink, Kempele, Finland). VO_2peak_ was considered as the highest 15-s mean value. The test ended at the participants’ voluntary exhaustion and the results were considered as VO_2peak_ if two of the following criteria were met: failure of VO_2_ to increase with increased exercise intensity; RER ≥1.1; maximal HR (HR_max_) ≥85% of age-predicted HR_max_ [40]. The age-predicted HR_max_ was determined by the formula 208-(age x 0.7) [41]. Aerobic performance was evaluated by the YYIE1. The YYIE1 test was performed on the same indoor TH wooden floor court as the matches, after a 10-min warm-up consisting of running at different speeds and changes of direction. The test consists of 2x20 m shuttle runs with increasing speeds interspersed by 5 s of active recovery, with the participants walking around a cone placed 2.5 m behind the starting/finishing line. At set intervals, the running speed increases, starting at 8.0 km⋅h^-1^. The total distance (m) covered during the test was recorded as test result for each participant [42].

#### Internal load outcomes procedures

One hundred and fifty-three HR recordings from 17 participants during the three game formats (9 matches per participant; 3 for each game format) were analyzed. Exercise intensity was assessed using HR monitors (Firstbeat Technologies Ltd., version 4.5.0.2, Jyväskylä, Finland). Selected HR zones were ≤60, 61–70, 71–80, 81–90, 91–100% HR_max_. In this study, the individual HR_max_ was determined as the highest value reached either during the VO_2max_ test, YYIE1 or matches, according to a multiple testing approach [43]. Capillary blood samples (30 μl) were drawn from the right earlobe to determinate BL concentrations (306 records from 17 players), at baseline (resting conditions) and at the end of the first and third period of the matches. For this analysis, a portable electroenzymatic lactate device analyzer (Lactate Pro 2 LT-1730, Arkray, Amsterdam, The Netherlands) was used. RPE is a practical, reliable and valid tool to estimate internal load and adding differential RPE (i.e., respiratory and muscular), may increase the sensitivity of internal load measurements [44]. Therefore, differential RPE [45] and fun levels (using a visual analogic scale; 0–10 AU) [46] were registered at the end of all game formats. Participants were familiarized with the use of the considered psychometric scales in training sessions performed before this study.

#### External load outcomes procedures

Video recordings (153 evaluations; 17 participants) (SONY-DCR-SX65E, digital video camera recorder, Weybridge, United Kingdom) were collected for activity profile characterization using time-motion analyses. Players’ displacements were divided into eight locomotor categories: 1) standing still, 2) walking, 3) jogging, 4) fast running, 5) sprinting, 6) sideways medium-intensity, 7) sideways high-intensity and 8) backwards movement. High-intensity movements were the result of the sum of fast running, sprinting and sideways high-intensity categories [21]. Individual speed thresholds were determined in order to account for the individual nature of the exercise intensity in each locomotor category [47]. For this purpose, each participant was instructed to perform each locomotor category at their individual speed, twice, over a 20 m distance, with 90 s of recovery in-between. Telemetric photoelectric cells (Brower Timing System, IRD-T175, Utah, USA) registered the individual speeds. The distance covered in each category was calculated by multiplying each participants’ individual speeds by the time spent in each locomotor category. This study participants mean speeds in each locomotor category were 0 km⋅h^-1^ for standing, 6 km⋅h^-1^ for walking, 9 km⋅h^-1^ 1 for jogging, 12 km⋅h^-1^ for fast running, 17 km⋅h^-1^ for sprinting, 8 km⋅h^-1^ for sideways medium-intensity, 10 km⋅h^-1^ for sideways high-intensity and 8 km⋅h^-1^ for backwards movements. Frequency of the selected high-demanding match actions, i.e., jumps, throws, stops, changes of direction and one-on-one situations, and total number of actions were registered via video-analysis of the matches.

Accelerometer data was collected using Catapult MinimaxX S4 (MinimaxX S4; Catapult Sports, Canberra, Australia) in indoor mode with global positioning system units (GPS) technology in inactive state. Data was downloaded and processed using Catapult sprint Version 5.1.1 (Catapult Innovations, Canberra, Australia). Units were located in a specific vest on players’ upper back. The validity and reliability of the accelerometers have been described elsewhere [48]. Player load (PL) (an estimate of physical demand combining the instantaneous rate of change in acceleration in 3 planes [49]) variables were evaluated at a 100 Hz sampling rate. In this study, PL was presented as percentage of time spent in PL zones 0–0.1, ˃0.1–0.3, ˃0.3–0.6, ˃0.6–1.0, ˃1.0–1.5, ˃1.5–2.0, ˃2.0 [49] and total accumulated PL. The matches were held under neutral temperature (20–22°C) and humidity conditions (50–60%).

### Statistical analysis

Data was tested for normal distribution using Shapiro-Wilk test. Results are presented as means ± standard deviations (SD). Differences between game formats’ internal and external load variables were assessed by repeated measures analysis of variance (ANOVA) with Bonferroni post hoc test for multiple comparisons tests. Power calculations were performed to detect an effect size of 0.25 in a one-way ANOVA of repeated measures (within subjects). Using 3 groups and 3 measurements, with correlation between measures of 0.75, alpha of 5%, and power of 80%, 15 participants were needed. Practical significance was assessed by calculating Cohen *d* and interpreted as trivial (<0.2), small (0.2–0.5), medium (0.5–0.8) and large (>0.8) [50]. IBM Statistical Package for the Social Sciences (SPSS), Statistics for Windows, (Version 25.0, Armonk, New York, USA: IBM Corp.) was used for all analyses. Statistical significance was set at *p*≤0.05.

## Results

### Internal load and fun levels during each game format

**Fig 2:**
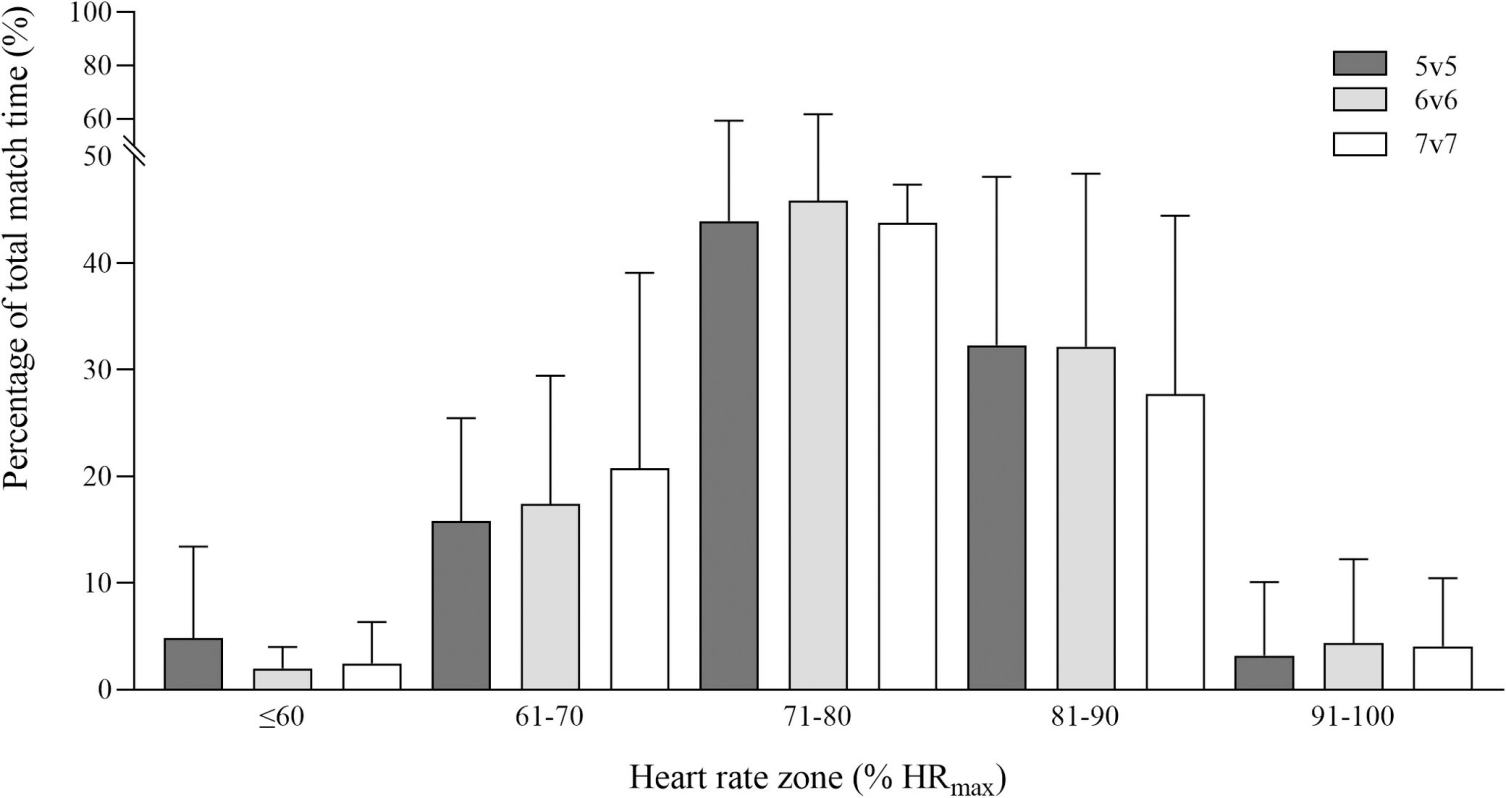
Percentage of total match time spent in each intensity zone expressed as percentage of players’ maximal heart rate (HR_max_) during 5v5 (dark grey bars), 6v6 (light grey bars) and 7v7 (white bars) recreational team handball game formats (data are presented as means ± SD).

Players’ internal load and fun variables for each game format (5v5, 6v6 and 7v7) are presented in Table 1 and Fig 2. No significant differences were found between game formats’ cardiovascular demands, RPE and BL, except for peak BL, which was significantly higher in 5v5 (5.6±2.1 mmol·l^-1^) than in 7v7 (4.7±1.7 mmol·l^-1^; *p =* 0.014, 95% CI: -1.4, -0.3, large).

**Table 1:** Players’ cardiovascular load, perceived experience, and fun levels during 5v5, 6v6 and 7v7 recreational team handball game formats (data are presented as means ± SD).

Variable	Game formats		
	5v5	6v6	7v7
**Heart rate**			
Mean HR (b·min^−1^)	129±9	129±10	128±11
Mean HR (%HR_max_)	77±5	77±4	76±6
Peak HR (b·min^−1^)	145±10	144±12	142±12
Peak HR (%HR_max_)	86±5	85±5	84±6
Time ˃80%HR_max_ (%)	35±19	37±21	32±21
Time ≤60%HR_max_ (%)	5±9	2±2	2±4
Time 61–70%HR_max_ (%)	16±10	17±12	21±18
Time 71–80%HR_max_ (%)	44±15	46±16	44±15
Time 81–90%HR_max_ (%)	32±16	32±16	28±17
Time 91–100%HR_max_ (%)	3±7	4±8	4±6
**Blood lactate**			
Match mean blood lactate (mmol·l^-1^)	3.9±1.5	3.7±1.3	3.6±1.4
Match peak blood lactate (mmol·l^-1^)	5.6±2.1	5.0±1.9	4.7±1.7
			*p = 0.014; *d =* 0.843
**Perceived experience**			
Respiratory RPE (AU, 0–10)	6.6±2.3	6.4±2.1	6.4±2.1
Muscular RPE (AU, 0–10)	6.6±2.4	6.3±2.1	6.1±2.1
Global RPE (AU, 0–10)	6.7±2.4	6.3±2.1	6.3±2.1
Fun (AU, 0–10)	9.0±1.0	9.0±0.9	8.6±1.6

HR–Heart rate; HR_max_−Maximal heart rate; RPE—Rating of perceived exertion; AU–Arbitrary units.

* Significantly different from 5v5.

**Fig 3:**
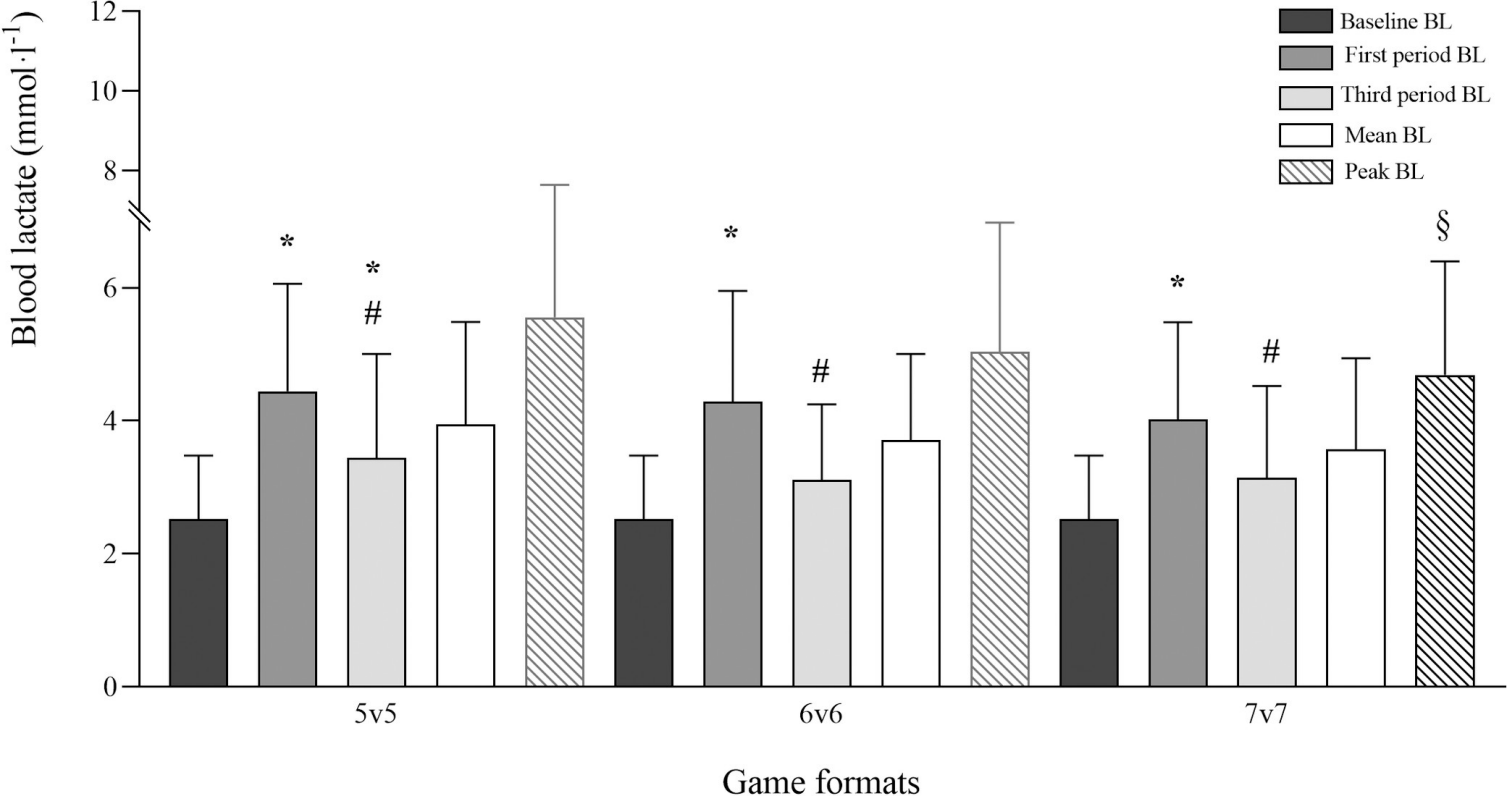
Baseline (dark grey bars), first period (medium grey bars), third period (light grey bars) mean blood lactate and match mean (white bars) and peak (listed bars) blood lactate levels during 5v5, 6v6 and 7v7 recreational team handball game formats (data are presented as means ± SD). BL–Blood lactate. * Significantly different from baseline; ^#^ significantly different from the first period and ^§^ significantly different from 5v5 (*p<*0.05).

Players’ BL values during 5v5, 6v6 and 7v7 game formats are presented in Fig 3. In all game formats, mean BL values increased significantly from baseline to the first period (5v5: *p<*0.001, 95% CI: 1.2–2.7, large; 6v6: *p≤*0.001, 95% CI: 1.0–2.6, large; 7v7: *p≤*0.001, 95% CI: 0.8–2.2, large) and decreased significantly from the first to the third period (5v5: *p≤*0.001, 95% CI: -1.5,-0.6, large; 6v6: *p≤*0.001, 95% CI: -1.7,-0.6, large; 7v7: *p≤*0.001, 95% CI: -1.3,-0.5, large).

### Activity profile during each game format

**Fig 4:**
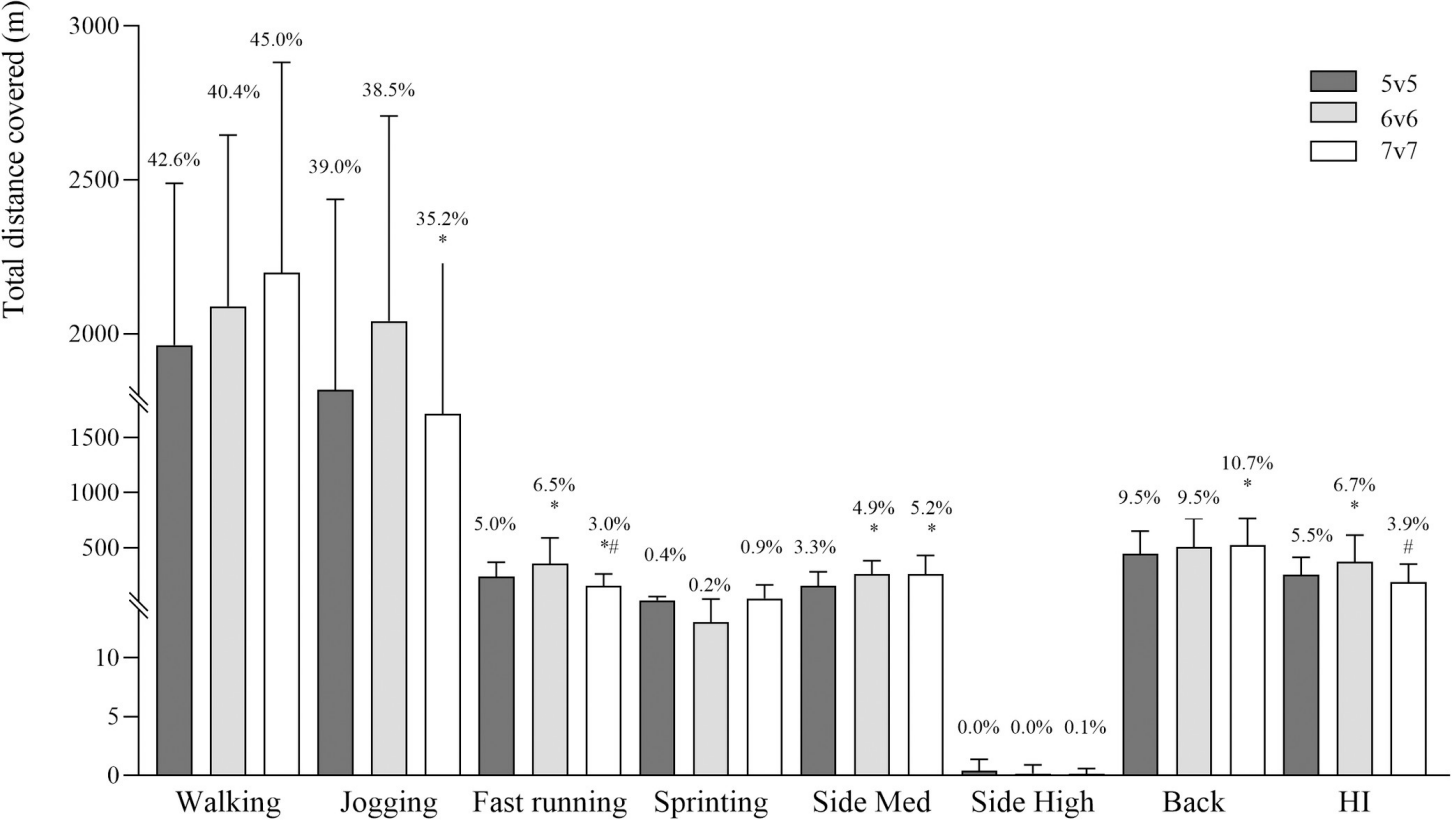
Total absolute and relative distance covered in the selected locomotor categories during 5v5 (dark grey bars), 6v6 (light grey bars) and 7v7 (white bars) recreational team handball game formats (data are presented as means ± SD). Side Med–sideways medium-intensity movements; Side High–sideways high-intensity movements; Back–backwards movements; High-intensity–sum of fast running, sprinting and sideways high-intensity movements. * Significantly different from 5v5 and ^#^ significantly different from 6v6 (*p≤*0.05).

Players’ locomotor profile during 5v5, 6v6 and 7v7 game formats is presented in Table 2. During 7v7 game format, the frequency of walking was significantly lower than 5v5 (*p≤*0.001, 95% CI: 1.3–3.6, large) and 6v6 (*p≤*0.001, 95% CI: 1.8–4.7, large). Additionally, 6v6 percentage of time spent jogging (*p≤*0.001, 95% CI: -5.1, -1.4, large), and 5v5 and 6v6 total distance covered (*p =* 0.004, 95% CI: -5.9, -1.7, large; *p =* 0.034, 95% CI: -5.7, -0.8, large; respectively; Fig 4) were significantly higher than 7v7. During 5v5 and 6v6, frequency (*p<*0.001, 95% CI: -9.3, -3.7, large; *p≤*0.001, 95% CI: -13.9, -5.4, large; respectively), percentage of time spent (*p =* 0.007, 95% CI: -1.7, -0.4, large; *p<*0.001, 95% CI: -2.9, -1.3, large; respectively), and total distance covered (*p =* 0.011, 95% CI: -138.0, -31.8, large; *p<*0.001, 95% CI: -298.5, -111.5, large; respectively) in fast running were significantly higher than during 7v7.

**Table 2:** Players’ locomotor profile during recreational team handball game formats (5v5, 6v6 and 7v7) (data are presented as means ± SD).

Locomotor categories										
	Standing	Walking	Jogging	Fast running	Sprinting	Side Med	Side High	Back	High-intensity	Total
**Freq (n)**										
5v5	16±6	89±12	78±16	17±8	1.4±1.9	14±9	0.1±0.1	30±11	18±9	245±35
6v6	8±5 ^*^p<0.001; *d* = 1.880	84±15	76±16	20±10	0.8±1.0	18±7 *p = 0.038; *d* = 0.707	0.1±0.2	30±13	21±11	237±45
7v7	9±5 ^*^p<0.001; *d* = 1.303	92±10	72±14	10±6 *p<0.001; *d* = 1.236 ^#^*p≤*0.001; *d* = 1.325	0.4±0.9 *p = 0.021; *d* = 1.199	19±11 *p = 0.002; *d* = 1.066	0.0±0.1	33±11	11±7 *p<0.001; *d* = 1.332 ^#^*p≤*0.001; *d* = 1.301	236±28
**Freq (%)**										
5v5	7±3	37±4	32±4	7±3	0.5±0.7	6±4	0.0±0.0	12±4	7±3	
6v6	3±2 ^*^p<0.001; *d* = 1.627	36±5	32±4	8±4 *p = 0.035; *d* = 0.782	0.3±0.4	8±3 *p = 0.004; *d* = 0.994	0.0±0.1	13±4	8±4	
7v7	4±2 ^*^p<0.001; *d* = 1.683	39±3 *p≤0.001*; d* = 1.121 ^#^*p≤*0.001; *d* = 1.287	31±4	4±2 *p<0.001; *d* = 1.273 ^#^*p<*0.001; *d* = 1.524	0.2±0.4 *p = 0.018; *d* = 1.227	8±4 *p<0.001; *d* = 1.466	0.0±0.1	14±4 *p = 0.043; *d* = 0.669	4±3 *p<0.001; *d* = 1.406 ^#^*p<*0.001; *d* = 1.457	
**Mean duration (s)**										
5v5	17±6	13±3	9±2	4±1	2±2	4±1	0.3±0.7	6±2	6±2	
6v6	21±6	15±2	11±2 *p = 0.035; *d* = 0.691	5±1	1±1	6±1 *p<0.001; *d* = 1.628	0.0±0.1	7±1	6±2	
7v7	21±5 *p = 0.045; *d* = 0.662	14±2	9±1 ^#^*p* = 0.004; *d* = 1.088	4±1 ^#^*p* = 0.002; *d* = 1.058	1±1	5±1 *p = 0.002; *d* = 1.052	0.2±0.7	7±2	5±2 *p = 0.045; *d* = 0.661 ^#^*p* = 0.004; *d* = 0.962	
**Total duration (s)**										
5v5	249±88	1182±236	734±213	69±37	4±7	68±57	0.2±0.4	192±73	73±42	2498±107
6v6	181±126 *p = 0.040; *d* = 0.716	1226±237	808±232	103±64 *p = 0.005; *d* = 1.292	3±3	113±56 *p<0.001; *d* = 1.622	0.1±0.2	220±100	106±67 *p = 0.011; *d* = 1.019	2654±298
7v7	202±90 *p = 0.037; *d* = 0.685	1256±248	683±183 *p = 0.032; *d* = 0.768 ^#^*p* = 0.015; *d* = 0.823	44±30 *p = 0.009; *d* = 0.866 ^#^*p≤*0.001; *d* = 1.439	1±3	110±75 *p<0.001; *d* = 1.739	0.0±0.1	229±94 *p = 0.020; *d* = 0.824	45±32 *p = 0.007; *d* = 0.921 ^#^*p≤*0.001; *d* = 1.420	2526±234
**Total duration (%)**										
5v5	10±3	47±9	30±9	3±1	0.2±0.3	3±2	0.0±0.0	8±3	3±2	
6v6	7±4 *p = 0.009; *d* = 0.884	46±9	16±3 *p<0.*001*; *d* = 0.213	4±2 *p = 0.011; *d* = 0.985	0.1±0.1	4±2 *p<0.001; *d* = 1.199	0.0±0.0	8±4	4±2 *p = 0.030; *d* = 0.781	
7v7	8±4 *p = 0.021; *d* = 0.755	50±8	27±7 ^#^*p<0*.*001*; *d* = 0.950	2±1 *p = 0.007; *d* = 0.912 ^#^*p<*0.001; *d* = 1.725	0.1±0.1	4±3 *p<0.001; *d* = 1.634	0.0±0.0	9±4 *p = 0.037; *d* = 0.756	2±1 *p = 0.005; *d* = 0.969 ^#^*p<*0.001; *d* = 1.699	
**Mean distance (m)**										
5v5		22±5	24±6	15±4	7±8	10±3	0.6±1.5	15±5	22±9	
6v6		25±6	27±7 *p = 0.037; *d* = 0.692	18±5	5±6	14±4 *p<0.001; *d* = 1.613	0.1±0.2	16±5	23±8	
7v7		24±7	23±4 ^#^*p* = 0.006; *d* = 1.171	14±4 ^#^*p* = 0.002; *d* = 1.046	3±5	12±4 *p = 0.005; *d* = 0.963	0.6±1.7	16±6	17±9 ^#^*p* = 0.002; *d* = 1.009	
**Total distance (m)**										
5v5		1962±525	1816±619	237±131	20±36	154±127	0.4±1.0	443±207	257±156	4632±617
6v6		2088±557	2041±666	357±231 *p = 0.007; *d* = 1.224	13±18	258±125 *p<0.001; *d* = 1.592	0.2±0.7	504±260	370±243 *p = 0.020; *d* = 0.906	5260±768 *p = 0.005; *d* = 0.927
7v7		2198±683	1713±524 *p = 0.010; *d* = 0.571	152±109 *p = 0.011; *d* = 0.838 ^#^*p≤*0.001; *d* = 1.414	37±123	258±170 *p<0.001; *d* = 1.524	0.1±0.4	524±240 *p = 0.033; *d* = 0.725	189±163 ^#^*p<*0.001; *d* = 1.688	4883±795
**Total distance (%)**										
5v5		43±12	39±12	5±3	0.4±0.8	3±3	0.0±0.0	9±4	5±3	
6v6		40±11	38±10	6±4 *p = 0.032; *d* = 0.777	0.2±0.3	5±2 *p≤0.001; *d* = 1.067	0.0±0.0	10±5	7±4	
7v7		45±11 ^#^*p* = 0.007; *d* = 0.880	35±9 *p = 0.004; *d* = 1.178 ^#^*p* = 0.034; *d* = 0.717	3±2 *p = 0.003; *d* = 1.017 ^#^*p<*0.001; *d* = 1.694	0.9±2.9	5±3 *p<0.001; *d* = 1.368	0.0±0.0	11±5	4±4 *p = 0.034; *d* = 0.699 ^#^*p<*0.001; *d* = 1.100	

Freq–Frequency; Side Med–sideways medium-intensity movements; Side High–sideways high-intensity movements; Back–backwards movements; High-intensity–sum of fast running, sprinting and sideways high-intensity movements.

* Significantly different from 5v5 and ^#^ significantly different from 6v6.

During 7v7 game format, high-intensity movements’ frequency was significantly lower than in 5v5 (*p<*0.001, 95% CI: -10.6, -4.3, large) and 6v6 (*p≤*0.001, 95% CI: -14.6, -5.5, large). During 5v5 and 7v7, percentage of time spent (*p =* 0.030, 95% CI: 0.3–1.7, large; *p<*0.001, 95% CI: -3.0, -1.3, large; respectively), and total distance covered (*p =* 0.020, 95% CI: 36.2–189.5, large; *p<*0.001, 95% CI: -249.0, -113.3, large; respectively) in high-intensity movements were significantly lower than during 6v6. Moreover, 5v5 percentage of time spent in high-intensity movements was significantly higher than 7v7 (*p =* 0.005, 95% CI: -1.8, -0.5, large). Players’ high-intensity actions frequency during the matches is presented in the Table 3. During 5v5 and 6v6, the number of throws (*p<*0.001, 95% CI: -4.7, -2.1, large; *p =* 0.031, 95% CI: -2.3, -0.4, large; respectively), stops (*p =* 0.017, 95% CI: -4.0, -0.8, large; *p =* 0.002, 95% CI: -3.3, -1.1, large; respectively) and total actions (*p =* 0.003, 95% CI: -13.2, -4.0, large; *p =* 0.017, 95% CI: -9.1, -1.8, large; respectively) was significantly higher than during 7v7 game formats.

**Table 3:** Players’ high-intensity game actions (data are presented as means ± SD) during 5v5, 6v6 and 7v7 recreational team handball game formats.

Actions	5v5	6v6	7v7
Jumps (n)	7.6±4.5	7.2±3.6	6.3±3.6
Throws (n)	8.7±3.8	6.6±3.4 *p = 0.003; *d* = 1.001	5.3±2.6 *p<0.001; *d* = 1.547 ^#^*p* = 0.031; *d* = 0.779
Stops (n)	12.6±4.0	12.4±4.0	10.2±3.2 *p = 0.017; *d* = 0.793 ^#^*p* = 0.002; *d* = 1.102
Changes of direction (n)	11.7±3.2	12.0±4.1	10.7±3.5
One-on-one situations (n)	8.8±2.9	8.0±2.2	8.2±1.8
Total actions (n)	49.3±15.1	46.2±14.4	40.7±11.9 *p = 0.003; *d* = 1.025 ^#^*p* = 0.017; *d* = 0.824

* Significantly different from 5v5 and

^#^ significantly different from 6v6.

During 5v5, 6v6 and 7v7 game formats, players’ percentage of time spent in 0.0–0.1, ˃0.1–0.3, ˃0.3–0.6, ˃0.6–1.0, ˃1.0–1.5, ˃1.5–2.0 and above 2.0 PL zones were 17–19%, 40–41%, 16–17%, 8%, 10–11%, 5–6% and 1%, respectively, and the total PL accumulated during the matches ranged between 288 to 310. The number of low, medium, high, and total accelerations during the game formats, ranged between 13–17, 7–9, 9–11 and 29–36 and the number of low, medium, high, and total decelerations ranged between 8–10, 4–5, 2–4 and 14–18, respectively. No significant differences were found between the game formats in PL zones and in low, medium, high, and total accelerations and decelerations variables.

### Differences between match periods

During 5v5, absolute and relative mean HR increased from the first to the second periods (*p =* 0.003, 95% CI: 1.5–4.9, large; *p =* 0.003, 95% CI: 0.9–3.0, large; respectively), remaining unaltered in the third period (Table 4). Absolute and relative mean HR, in 6v6 (*p =* 0.035, 95% CI: -6.2, -0.9, medium; *p =* 0.034, 95% CI: -3.6, -0.5, medium; respectively) and 7v7 (*p =* 0.010, 95% CI: -9.2, -2.2, large; *p =* 0.008, 95% CI: -5.4, -1.4, large; respectively), decreased as the match time progressed, showing significant differences between the first and third periods (Table 4). During 7v7 game format time spent above 80%HR_max_ significantly decreased from the first to the second (*p =* 0.027, 95% CI: -22.3, -3.7, medium) and third (*p =* 0.005, 95% CI: -27.3, -7.9, large) period, and from the second to the third period (*p =* 0.027, 95% CI: -11.4–2.0, medium).

**Table 4:** Players’ mean, peak and percentage of total match time spent in the different heart rate zones during the three match periods in 5v5, 6v6 and 7v7 recreational team handball game formats (data are presented as means ± SD).

Variables	Game periods								
	5v5			6v6			7v7		
	1^st^ period	2^nd^ period	3^rd^ period	1^st^ period	2^nd^ period	3^rd^ period	1^st^ period	2^nd^ period	3^rd^ period
Mean HR (b·min^−1^)	127±8	130±9 *p = 0.003; *d* = 1.017	129±9	131±10	130±12	127±11 *p = 0.035; *d* = 0.692	131±9	127±13	125±11 *p = 0.010; *d* = 0.855
Mean HR (%HR_max_)	76±4	77±5 *p = 0.003; *d* = 1.124	77±5	77±4	77±5	75±5 *p = 0.034; *d* = 0.730	78±5	76±6	74±6 *p = 0.008; *d* = 0.872
Peak HR (b·min^−1^)	145±10	146±10	144±10	147±12	145±12	141±14 *p≤0.001; *d* = 1.142 *p = 0.017; *d* = 0.819	145±11	142±14	139±13 *p = 0.026; *d* = 0.736
Peak HR (%HR_max_)	86±6	87±6	85±5	87±5	86±5	84±6 *p≤0.001; *d* = 1.247 *p = 0.014; *d* = 0.833	86±6	84±7	83±7 *p = 0.020; *d* = 0.772
Time >80%HR_max_ (%)	33±18	40±23	34±23	39±22	41±23	30±26	42±26	29±24 *p = 0.027; *d* = 0.717	24±21 *p = 0.005; *d* = 0.950 *p = 0.027; *d* = 0.368
Time ≤60%HR_max_ (%)	7±7	4±10	3±10 *p = 0.045; *d* = 0.734	3±3	1±2 *p = 0.008; *d* = 0.892	2±2	3±5	2±3	2±5
Time 61–70%HR_max_ (%)	18±12	14±10	16±11	13±9	14±11	25±23	17±16	21±21	24±20 *p = 0.023; *d* = 0.799
Time 71–80%HR_max_ (%)	42±15	42±18	48±18	46±19	42±19	49±20	40±17	45±20	46±16
Time 81–90%HR_max_ (%)	31±15	35±20	30±19	35±17	36±21	26±18 *p = 0.015; *d* = 0.799	37±22	24±17	22±18
Time 91–100%HR_max_ (%)	2±4	4±9	3±9	5±7	5±9	4±11	5±7	5±10	2±5

HR–Heart rate; HR_max_−Maximal heart rate.

* Significantly different from the first match period; ^#^significantly different from the second match period.

Total number of high-intensity game actions decreased from the first to the second period for 6v6 (*p =* 0.016, 95% CI: -6.6, -1.4, large) and 7v7 (*p =* 0.020, 95% CI: -4.1, -0.8, large), and from the first to the third period for 6v6 (*p<*0.001, 95% CI: -8.1, -3.8 large). Distance covered in fast running (*p<*0.001, 95% CI: -4.7, -2.5, large) and sprinting (*p =* 0.032, 95% CI: -0.5, -0.1, large) decreased from the first to the third period for 6v6.

## Discussion

The aim of this study was to describe the acute physiological response, activity profile and fun levels of 5v5, 6v6 and 7v7 recreational TH game formats in over 60-year-old men. This to provide physical exercise and sport professionals, evidence on the internal and external load characteristics of the game formats analyzed, that will allow them to make informed decisions according to the defined purposes for the training sessions. The main findings of this study were that game format had no significant impact on match internal load, although a tendency was observed for higher demands in 5v5 and 6v6 than in 7v7. Significant differences were evident in the external load variables, with 5v5 and 6v6, showing a higher number of high-intensity movements and total high-intensity game actions when compared to 7v7.

### Internal load and fun levels during the game formats

During recreational TH training sessions using SSGs, mean HR is typically reported to be within 76–85%HR_max_ [18, 21–26, 32]. In the present study, mean HR values for the three game formats were lower (76–77%HR_max_) than values observed in young adult men and women, premenopausal women and adult/middle-aged men (81–85%HR_max_) [18, 24, 25], but equal to or higher than those reported for postmenopausal women (76%HR_max_) enrolled in a TH intervention study that resulted in cardiovascular improvements [23]. In fact, these values are in the range of the vigorous exercise intensity threshold (60–85%HR_max_) proposed to promote cardiovascular improvements [33].

Peak HR values were lower than those reported in studies using recreational soccer SSGs with elderly males (84–86 vs 99%HR_max_, respectively) and, consequently, the percentage of time spent above 90% HR_max_ was also lower (4 vs 48% of total match time) [31]. However, studies using recreational floorball [51, 52] and recreational TH [23] with similar age groups showed results in line with our study. Additionally, it is worth noting that in the present study we assessed the participants’ HR_max_ making use of a multiple approach in order to have an as accurate as possible value [43]. Having an accurate HR_max_ is of great practical interest as time spent above 90%HR_max_ was reported to be related to improvements in cardiorespiratory fitness in recreational TH [18]. Given that, the above differences in exercise HR may be the result of unsuitable HR_max_ assessment [15].

No significant differences were found in exercise HR between the three game formats. This is in accordance with a recent study comparing 4v4, 5v5 and 6v6 game formats using the same pitch size (40x20 m) for young (20.8±1.1 years) active college students with no competitive experience in TH [32]. However, our study reported a significant decrease in 6v6 and 7v7 game formats’ mean and peak HR in the third comparing to the first match period, while mean HR in 5v5 significantly increased from the first to the second period and was then maintained during the last 15-min period of the matches. This decrease in intensity was also shown in the activity profile. The main relevance of these results is that 5v5 game format seems to be more efficient in maintaining the cardiovascular load throughout 45-min matches, perhaps due to the greater involvement in the game imposed by the lower number of players. Nonetheless, exercise intensity during all match duration in the three game formats was within the range (60–85%HR_max_) proposed for cardiovascular improvements [33].

The practical implication of this study HR results is that they were in line with the results from other studies that used recreational team sports, especially TH, that reported cardiovascular improvements after 12-16-week interventions. Thus, future studies should address the role of training volume and intensity on practitioners’ health and fitness.

In this study, peak BL was significantly lower in 7v7 than in 5v5, meaning that in 5v5, the participants, may achieve higher anaerobic intensities. Mean and peak BL concentrations (3.6±1.4 and 4.7±1.7 mmol·l^-1^) were in line with the values reported in former TH players, during 7v7 matches (3.6±1.3 and 4.2±1.2 mmol·l^-1^) [21]. Significantly higher values were found in all game formats in the first period comparing to baseline conditions and in 5v5, in the third period comparing to baseline. This is in line with a study involving former TH players playing recreational matches [21]. Additionally, a decrease in BL values was found from the first to the third period in all game formats, which again, is in line with what has been shown for recreational TH players with previous experience with the sport [21], evidencing an intensity decrease from the beginning to the end of the recreational TH matches. This decrease is in accordance with the mean and peak HR decrease shown from the first to the third match period, as well as with the decrease in the number of high-intensity game actions during the match in 6v6 and 7v7 game formats, although not observed for 5v5.

In our study, no differences were found in RPE between game formats, with the intensity being perceived as strong-to-very-strong (6.1–6.7 AU). These values were lower than those reported for former TH players playing the recreational version of this sport [18]. However, higher than observed for postmenopausal women (4.8, AU, playing 5v5 and 6v6 matches) [23] and for male college students (3.9 AU, playing 6v6 matches) [32] performing recreational TH. The absence of differences in RPE when playing 5v5, 6v6 or 7v7 may be explained by participants’ lack of experience with this sport and by a spontaneous adjustment to game format demands as shown by exercise HR consistency across the proposed game formats. It is important to highlight the very high fun levels reported by the participants (9 out of 10) while playing recreational TH, since enjoyment is a key factor to increase motivation and assure long-term adherence to an exercise program [53]. Also, the perceived high rate of fun in this population may mask the high metabolic, musculoskeletal, and cardiorespiratory strain during training interventions.

### Activity profile during the game formats

Significant differences were found between the game formats’ external load, namely high-intensity locomotor activity variables. Standing frequency was significantly higher in 5v5 than in 6v6 and 7v7, which may be related to more stops being needed to recover since there are less players involved in the match. The walking frequency was significantly higher in 7v7 than in 5v5 and 6v6 and the jogging frequency showed no significant differences between the game formats. These variables were the ones in which the participants spent more time during the matches (76–77% of total match time) in all game formats. Furthermore, the frequency of fast running movements was significantly higher in 6v6 than in 5v5 and 7v7. Frequency of sprints, resulted higher in 5v5 than in 7v7. Moreover, the frequency of high-intensity movements was significantly higher in 5v5 and 6v6 than in 7v7. These results show that 5v5 and 6v6 may induce higher load on muscles and bones than 7v7.

Standing time was significantly higher in 5v5 than in 6v6 and 7v7. When comparing to former TH players playing the recreational version of the sport, our participants’ standing time was higher (8–10 vs 4%), which may be related to their lower physical fitness (27.9±4.1 vs 40.2±7.0 mL⋅min^-1^⋅kg^−1^). The 7v7 game format revealed to promote significantly more jogging and less high-intensity distance covered than the other game formats. Interestingly, match time spent with high-intensity movements (2–4%) was similar to that reported in elite (4%) and recreational TH players with previous experience in the sport (6%) [21]. Time spent at high-intensity seems to be a good indicator when the purpose is to achieve cardiovascular adaptations [13]. However, caution should be taken when comparing these results with those studies, due to differences in speed thresholds. The 5v5 game format showed a significantly higher number of throws and stops than 6v6 and 7v7, suggesting a greater individual game involvement due to the reduced number of players. The practical implication of these results is that 5v5 and 6v6 may be better options than 7v7, when the aim is to induce musculoskeletal improvements in this population. Moreover, when playing 5v5 and 6v6, the participants performed higher number of specific TH game actions, which leads to a higher participation in the training session, and consequently, may result in higher motivation.

We had hypothesized that 5v5 game format would elicit higher cardiovascular (internal load) and activity profile (external load) demands due to the larger playing area, and, consequently, lower player density, and higher fun levels as a result of higher participant involvement in the match. However, changing the number of players (5v5, 6v6 and 7v7) in the same pitch size (40x20 m) did not result in significant differences in majority of these variables. The practical application of these results is that all the three game formats elicited high loading in over 60-year-old inactive men and therefore, all can be recommended as options for organizing recreational TH.

In summary, this study results may guide physical exercise professionals and/or TH coaches, on the best practices when using recreational TH as exercise mode to promote physical fitness and health of older populations.

Although in this study we analyzed the game formats typically used in recreational TH-based exercise interventions (i.e., 5v5, 6v6 and 7v7), a study limitation is the fact that we did not study other game formats’ demands, namely 4v4 or 3v3, as there could be differences in internal and external load in comparison to other populations. Additionally, other contextual variables should be addressed in the future, namely court dimensions and comparing indoor vs outdoor pitches, as this type of exercise program may be implemented in different environments. Future studies with more participants may be used to elucidate whether there are some minor advantages in relation to intensity and fun scores by using 5v5 and 6v6 in comparison to 7v7, in a 40x20 m TH court, for over 60-year-old inactive men. Future research should also test the training effects of the proposed different recreational TH formats on participants’ health and physical fitness.

## Conclusions

Recreational TH internal load demands are similar either played as small-sided (5v5, 6v6) or formal game formats (7v7), in the same pitch size (40x20 m), and are within the range to induce cardiovascular adaptations. This, across match time periods (i.e., 3x15-min). Higher frequency of high-intensity game actions was found in 5v5 and 6v6. Accordingly, these game formats may be better options when the purpose is to induce musculoskeletal improvements in this population.

The higher number of total actions and throws found in 5v5 and 6v6 may also reveal to be of practical importance as a greater involvement may lead to a higher level of motivation and therefore, to higher fun levels and long-term adherence to the exercise program. Nonetheless, recreational TH practice is a highly motivational activity, whatever the chosen game format.

From a practical point of view, this study results suggest that recreational TH can be a valid exercise option to promote health improvements in over 60-year-old men.

A multiple game format approach may be used in recreational TH interventions to provide training variety and training sessions should last up to 60 min. Additionally, considering the very high fun levels reported during recreational TH matches and that lack of motivation to exercise is a major hurdle, future intervention studies using this exercise mode for this population are warranted.
